# Sensing Platform Based on Gold Nanoclusters and Nanoporous Anodic Alumina for Preeclampsia Detection

**DOI:** 10.3390/bios14120610

**Published:** 2024-12-13

**Authors:** Josep Maria Cantons, Akash Bachhuka, Lluis F. Marsal

**Affiliations:** 1 Department of Electronics, Electric, and Automatic Engineering, Rovira I Virgili University (URV), 43007 Tarragona, Spain; josepmaria.cantons@urv.cat; 2 Institute of Chemical Research of Catalonia (ICIQ), 43007 Tarragona, Spain

**Keywords:** nanoporous anodic alumina, gold nanoclusters, photoluminescence, biosensing, preeclampsia and endoglin

## Abstract

Preeclampsia is a pregnancy-specific hypertensive syndrome recognized as the leading cause of maternal and fetal morbidity worldwide. Early diagnosis is crucial for mitigating its adverse effects, and recent investigations have identified endoglin as a potential biomarker for this purpose. Here, we present the development of a hybrid biosensor platform for the ultrasensitive detection of endoglin, aimed at enabling the early diagnosis of preeclampsia. This platform integrates the high surface area properties of nanoporous anodic alumina (NAA) with the unique optical characteristics of gold nanoclusters (AuNCs) to achieve enhanced detection capabilities. The NAA surface functionalized to promote attachment of AuNCs, which then was functionalized with specific antibodies to confer selectivity towards endoglin. Photoluminescence (PL) analysis of the biosensor demonstrated a linear detection range of 10–50 ng/mL, with a detection limit of 5.4 ng/mL and a sensitivity of 0.004 a.u./(ng/mL). This proof-of-concept study suggests that the NAA-AuNCs-based biosensing platform holds significant potential for the development of ultrasensitive, portable, and cost-effective diagnostic tools for preeclampsia, offering a promising avenue for advancing prenatal care.

## 1. Introduction

Preeclampsia is a prevalent and serious complication of pregnancy that affects 5–8% of pregnant women globally and impacts approximately 8.5 million women annually [1,2]. This hypertensive disorder [3,4,5], which typically manifests after 20 weeks of gestation, is a leading cause of fetal, maternal, and infant mortality [6,7,8]. The pathophysiology of preeclampsia is complex, often involving placental hypoxia, proteinuria, endothelial dysfunction, end-organ ischemia, and increased vascular permeability. The resulting vasoconstriction of placental arteries can severely compromise blood flow—posing significant risks to both mother and baby [9]. It is estimated that preeclampsia contributes to approximately 42,000 maternal deaths worldwide each year [10]. Current diagnostic criteria for preeclampsia primarily rely on elevated arterial blood pressure measurements (≥140/90 mm Hg) after 20 weeks of gestation [11,12] and the presence of proteinuria (≥0.3 g in a 24-h urine sample) [13]. However, these methods often detect the condition only after significant pathological changes have occurred, underscoring the critical need for earlier and more precise diagnostic tools [14].

Recent advances in biomarker research have opened new avenues for the early detection of preeclampsia. For Instance, Chen et al. combined enzyme-linked immunosorbent assay (ELISA) with the plasmonic properties of gold nanoparticles to detect a tumoral inhibitor (CD81), a potential biomarker for preeclampsia [15]. Similarly, functionalized nanochannels have been employed to capture estradiol (E2), another molecule implicated in preeclampsia [16]. Numerous other biomarkers, such as preeclampsia-related RNA [17] and specific enzymes [18], have been investigated to enhance the specificity and sensitivity of diagnostic assays. Among these, endoglin (CD105), a glycoprotein in elevated levels in the blood plasma of women with preeclampsia, has emerged as a particularly promising target [19]. Clinical studies have demonstrated that endoglin levels exceeding 20 ng/mL are associated with a significantly increased risk of preeclampsia [20,21,22]. Furthermore, endoglin concentrations between 8 ng/mL and 10 ng/mL, depending on gestational age, have also been indicative of potential preeclampsia development.

Given the clinical importance of endoglin as a biomarker, there is an urgent need for a rapid, cost-effective biosensor that can facilitate early diagnosis of preeclampsia. In response to this need, we have developed an innovative hybrid biosensor platform, that integrates NAA with AuNCs, for the detection of endoglin. AuNCs are renowned for their exceptional optical properties, including strong fluorescence, which makes them ideal for various biomedical applications [23,24,25], such as imaging, detection, and therapeutic interventions [26,27]. The synthesized AuNCs, composed of more than 29 atoms and with sizes less than 2 nm, offer unique advantages in biosensing applications due to their excellent chemical and physical properties, as well as their biocompatibility [28].

NAA has been widely recognized for its remarkable geometric and surface properties, which make it highly suitable for sensing and photonic applications [29,30,31,32,33,34,35]. Its high effective surface area, which can reach hundreds of m^2^/cm^3^, allows for the integration of large quantities of AuNCs, thereby enhancing the exposure and interaction with target antibodies. The ability to manipulate the optical properties of alumina by adjusting fabrication parameters further expands its utility in various biosensing applications [36,37,38,39,40]. NAA has previously been employed in the detection of DNA fragments specific to salmonella [41], micro-RNAs specific to breast cancer [42], thrombin [43], and tumor necrosis factor-alpha [44].

The primary objective of this study was to develop a rapid and portable biosensor for early preeclampsia diagnosis. By leveraging the high surface area of NAA and the superior optical properties of AuNCs, we aim to create a highly sensitive platform for detection, offering significant potential for advancing prenatal diagnostics and improving maternal and fetal outcomes.

## 2. Materials and Methods

### 2.1. Materials

High-purity aluminium substrates (99.999%) with a thickness of 0.5 mm were purchased from Goodfellow Cambridge Ltd. (Cambridge, UK). Ethanol (C2H6O), acetone (C3H6O), oxalic acid (C2H2O4), perchloric acid (HClO4), chromic acid (H2CrO4), glutathione reduced (C10H17N3O6S), gold chloride III (AuCl3), Hydrogen peroxide (H2O2), 3-Aminopropyltriethoxysilane (C9H23NO3Si), 1-ethyl-3-(3-dimethylaminopropyl) carbodiimide (C8H17N3), N-hydroxy succinimide (C4H5NO3), Glucose Oxidase (GLU) from Aspergillus niger, Bovine Serum Albumin (BSA) and Human Serum Albumin (HSA) were supplied by Sigma Aldrich. Recombinant Human endoglin CD105 protein (ENG) and Recombinant Anti-CD105 antibody [EPR19911-220] (antiEndoglin) were procured from Abcam. Double de-ionized (DI) water (18.6 MΩ, Milli-Q^®^) was used for all the solutions unless otherwise specified.

### 2.2. Fabrication of Self-Ordered Nanoporous Anodic Alumina

Self-ordered nanoporous anodic alumina samples were fabricated by anodizing aluminium using a two-step anodization method [45,46]. To remove all the impurities and grease, aluminium (Al) foils were cleaned with acetone, DI water, and ethanol (EtOH). Before anodization, aluminium substrates were electropolished in a solution of ethanol-perchloric acid (HClO_4_) at 4:1 (*v*:*v*) at 20 V and 5 °C for 6 min. The first anodization step was conducted in a 0.3 M oxalic acid (H_2_C_2_O_4_) solution at 40 V and 5 °C for 20 h, resulting in the formation of disordered pores on the aluminum surface. As anodization progressed, the pores self-ordered at the substrate interface. The grown aluminium oxide was removed using a mixture of phosphoric acid (H_3_PO_4_) 0.4 M and chromic acid (H_2_CrO_4_) 0.2 M at 70 °C for 3 h. The resulting Al showed a surface with a highly ordered pattern formed by nanoconcavities.

A second anodization step was performed under the same conditions as the first to achieve the desired pore structure. The final pore length was controlled by adjusting the anodization charge, and samples were anodized to achieve a pore length of 6 µm by applying a charge of 95 C over a total anodized area of 6.28 cm^2^. To increase the diameter of pores (pore widening), wet chemical etching was performed in phosphoric acid (H_3_PO_4_) at 5% wt. at 35 °C for 20 min, at a rate of 1.2 nm/min. Lastly, the samples were washed with DI water and ethanol and then air-dried.

### 2.3. Synthesis of Gold Nanoclusters

Gold nanoclusters (AuNCs) were synthesized by mixing a 0.5 mL of 20 mM solution of gold (III) chloride trihydrate (HAuCl_4_·3H_2_O) with 0.15 mL of 100 mM solution of reduced glutathione (GSH) in 4.35 mL of Milli-Q water at 25 °C. The mixed solution was heated at 70 °C with continuous stirring at 500 rpm for 24 h. This process yielded AuNCs with an approximate size of 2 nm. To prevent aggregation, the pH of the AuNCs solution was adjusted to a basic value of 8.2.

### 2.4. APTES Functionalization

The NAA surface was functionalized with APTES to introduce cross-linked amino groups, facilitating the immobilization of biomolecules and enhancing biosensor selectivity and sensitivity [47,48]. Initially, surface hydroxylation was achieved by immersing the NAA samples in boiling hydrogen peroxide (H_2_O_2_) for 30 min, followed by drying in an oven at 60 °C for 1 h. Subsequently, to attach silane and amine groups, the hydroxylated samples were treated with a solution of 9.5 mL toluene and 0.5 mL of 5% APTES for 1 h, followed by curing in an oven at 100 °C.

### 2.5. Gold Nanoclusters Surface Activation

The AuNCs were activated using a 1:1 mixture of 0.05 M EDC and 0.1 M NHS, which facilitates the activation of carboxyl groups on the AuNCs, enabling their binding to the amine-functionalized NAA surface. The activated AuNCs solution (500 µL) was incubated with the functionalized NAA surface for 1 h under gentle agitation. The samples were then rinsed with DI water for 15 min (see Figure S1 for process schematic).

### 2.6. Antibody Functionalization

**Figure 1:**
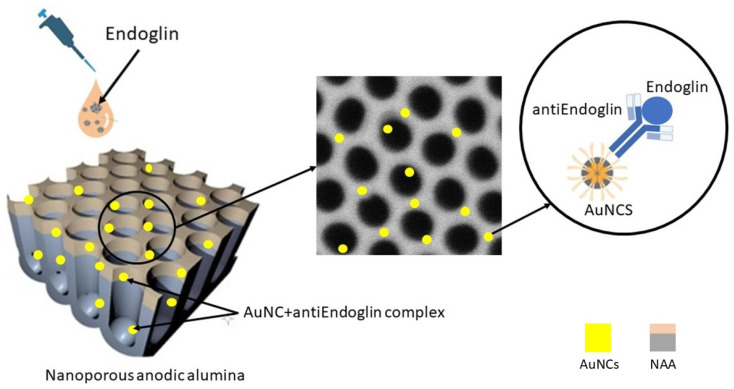
The schematic illustration shows the behaviour of the antiEndoglin– Endoglin hybrid biosensor.

To test the biosensor, anti-Endoglin antibodies specific to endoglin were immobilized on the gold-coated surfaces. Human serum albumin (HSA) and glucose oxidase (GLU) were used as control proteins to verify biosensor selectivity. The functionalized surfaces were incubated with a 5.39 µM anti-Endoglin antibody solution for 1 h with gentle shaking. Post-incubation, the surfaces were blocked with bovine serum albumin (BSA) blocking buffer for 30 min to prevent non-specific protein binding [49,50,51]. The samples were then rinsed three times with PBS and dried using N_2_ gas. Figure 1 provides a schematic overview of the biosensor, depicting the attachment of anti-Endoglin to the AuNCs and the subsequent capture of endoglin.

### 2.7. Field Emission Scanning Electron Microscope (FESEM) Characterization

Scanning electron microscopy images were performed with a Field Emission Scanning Electron Microscope (FESEM) (Scios 2, Thermo Fisher Scientific, Waltham, MA, USA) at a chamber pressure of 1 × 10^−4^ Pa with electron beam voltages set between 5 kV, and at magnifications of 50 KX to 250 KX, depending on the sample. The Everhart-Thornley detector (ETD) was used to detect secondary electrons (SE).

### 2.8. FTIR Characterization

FTIR measurements were performed in the transmittance mode with a Jasco FT/IR-6700, infrared spectrometer (Bruker Española S.A, Madrid, Spain) in the midinfrared region from 4000 to 450 cm^−1^, with a resolution of 4.0 cm^−1^ and 64 scans.

### 2.9. Photoluminescence Characterization (PL)

Photoluminescence (PL) characterization of the AuNC-modified NAA samples was performed using a Photon Technology International Inc. spectrophotometer (Division of Horiba, Irvine, California, USA) equipped with a xenon (Xe) lamp as the excitation source. Measurements were taken at room temperature with an excitation wavelength of λex = 350 nm. The excitation and emission slits were set to an aperture of 5 nm at the beam entrance and exit.

### 2.10. HRTEM Characterization

High-resolution TEM JEOL F200 (HRTEM) (JEOL (Europe) B.V, Nieuw-Vennep, Netherlands) was used to characterize the gold nanoclusters. The device worked in scanning mode with a beam energy of 200 kV, and the magnification used was 10 MX.

## 3. Results and Discussion

### 3.1. Surface Characterization

**Figure 2:**
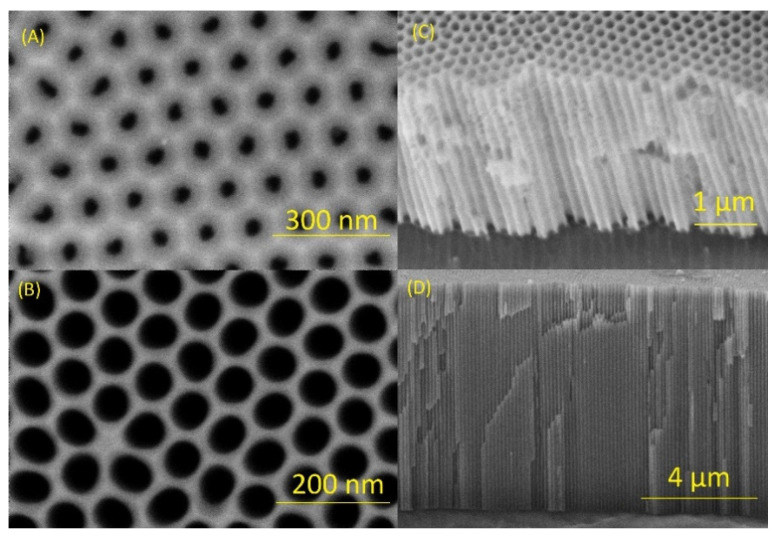
FESEM images of nanoporous anodic alumina showing (**A**) top-view of a self-ordered NAA sample with a size of 32 ± 3 nm, (**B**) top-view of a self-ordered NAA sample after pore widening treatment with a pore size of 65 ± 4 nm, (**C**) cross-section of NAA sample which shows its nanoporous structure with a thickness of 5.1 ± 0.2 μm, (**D**) cross-section of NAA sample which shows a thickness of 6.0 ± 0.2 μm.

NAA images from both the top-view and cross-section views were acquired using FESEM to validate the fabrication process and characterize the geometrical features of the samples. Figure 2A and Figure S3 shows a top-view of NAA with a pore diameter of 32 ± 3 nm and an interpore distance of 101 ± 2 nm. To increase the pore diameter, a wet chemical etching process was applied, resulting in an enlarged pore diameter of 65 ± 4 nm and an interpore distance of 102 ± 3 nm, as depicted in Figure 2B and Figure S4. The honeycomb-like arrangement and uniform interpore distances confirm the high degree of order achieved in the NAA structure. Cross-sectional FESEM images (Figure 2C,D) further illustrate the cylindrical and parallel nature of the pores, with lengths of Lp = 5.1 ± 0.2 μm and Lp = 6.0 ± 0.2 μm, respectively. These images also reveal the hexagonal arrangement of pores on the upper surface, indicative of the high precision in the NAA fabrication process.

### 3.2. Characterization of Gold Nanoclusters

**Figure 3:**
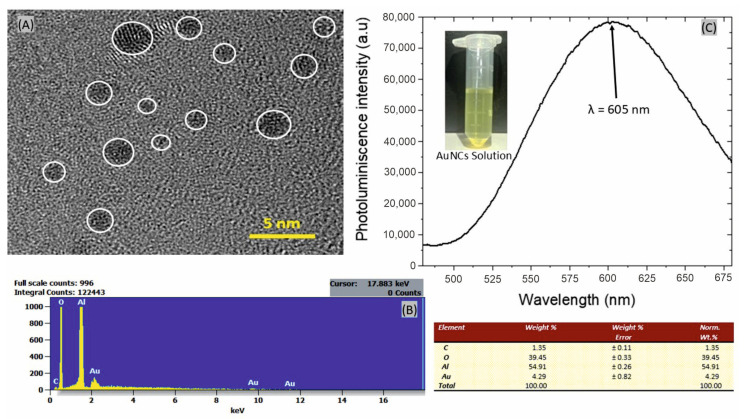
Characterization of AuNCs showing (**A**) an HRTEM image that shows the synthesized gold nanoclusters with a cluster size of 1.4 ± 0.3 nm. A white line surrounding the gold nanoclusters is included to define the border better, (**B**) shows an EDS analysis of a NAA-AuNCs functionalized structure to demonstrate the presence of the nanoclusters, (**C**) shows a Photoluminescence spectrum of an AuNCs solution excited at λexc = 350 nm. Inset photography of AuNCs (20 μM) under visible light.

High-resolution transmission electron microscopy (HRTEM), energy-dispersive x-ray spectroscopy (EDS), and photoluminescence spectroscopy were used to evaluate the physicochemical properties of gold nanoclusters (AuNCs). Figure 3A shows a HRTEM image of the synthesized gold nanoclusters, which have an average size of 1.4 ± 0.3 nm. Subsequently, an EDS analysis of the functionalized NAA samples was performed to confirm the attachment of AuNCs to the nanostructure, revealing a weight value of Au at 4.29% (Figure 3B). The fluorescence behavior of gold nanoclusters in solution was investigated utilizing a photoluminescence (PL) spectrophotometer, with excitation occurring at a wavelength of 350 nm. The PL spectrum (Figure 3C) exhibits a distinct emission peak at 600 nm, characteristic of AuNCs with a size of 2 nm or less. This photoluminescent behavior arises from the quantum confinement effect, which results in the discretization of energy levels due to the breakdown of the continuous band structure [52]. The PL emission, driven by intraband (sp-sp) and interband (sp-d) transitions, mirrors the behavior observed in organic dye molecules, where electronic transitions between energy levels result in light absorption and emission [53].

### 3.3. Surface Functionalization and FTIR Analysis

**Figure 4:**
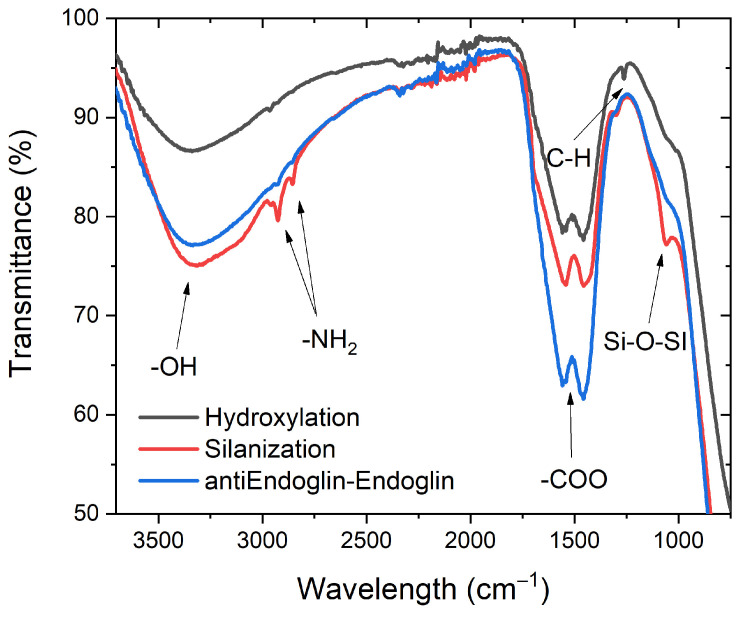
FTIR spectra of NAA samples in different stages of functionalization.

NAA substrates underwent a series of surface modifications involving hydroxylation and functionalization using 3-aminopropyltriethoxysilane (APTES) to introduce amine groups, thereby enabling the attachment of carboxyl-capped gold nanoclusters. The progression of functionalization stages, including hydroxylation, silanization, and antibody-endoglin conjugation, was validated through Fourier-transform infrared spectroscopy (FTIR). The FTIR spectrum in Figure 4 depicts distinct features at various wavenumbers, affirming the successful evolution of NAA during each functionalization step. The broad absorption band around 3300 cm⁻^1^ corresponds to the stretching vibrations of hydroxyl groups (-OH) from alumina and hydrogen peroxide [54,55], indicative of the Al–OH bond [56]. The intense doublet absorption peaks at 1459 cm⁻^1^ and 1555 cm⁻^1^ are attributed to the symmetric and asymmetric stretching of carboxylic groups (-COO), respectively [57,58,59]. A minor signal at 1264 cm⁻^1^ is associated with the C-H bond from residual oxalic acid [60]. The absorption peak at 1054 cm^−1^ confirms the formation of Si–O–Si links from APTES [61], while additional peaks observed at 2854 cm^−1^ and 2925 cm^−1^ correspond to the presence of -NH2 groups [62]. Following protein binding, a notable reduction in -NH2 peaks suggests successful attachment of gold nanoclusters and/or antibody-endoglin complexes to the modified surface.

### 3.4. Photoluminescence Stability and Biosensor Performance

The PL stability of gold nanoclusters immobilized on APTES-NAA samples was examined under two conditions: (a) with NHS/EDC coupling and (b) without NHS/EDC coupling. NHS/EDC coupling is a well-known cross-linking strategy that activates carboxyl groups on the AuNCs, facilitating their covalent attachment to the amine-functionalized NAA surface. This chemical conjugation forms stable amide bonds, which are crucial for maintaining the integrity and stability of the immobilized nanoclusters. As shown in Figure S2, samples subjected to NHS/EDC coupling exhibited significantly enhanced and more consistent PL intensity, indicating that this coupling method prevents nanocluster aggregation and degradation, which could otherwise quench the photoluminescence. The covalent bonding ensures that the AuNCs remain securely anchored to the NAA surface, preserving their quantum confinement properties that are responsible for their strong PL emission.

To optimize the biosensor’s performance, various incubation times were also tested for the efficient capture of endoglin on the antibody-functionalized NAA surface. Optimal PL response was achieved with a 15-min incubation period followed by a 5-min PBS wash. This incubation allows sufficient time for the specific binding of endoglin to the anti-endoglin antibodies, which are conjugated to the AuNCs. To reduce nonspecific binding, the antibody-coated NAA substrates were blocked with bovine serum albumin (BSA) at concentration of 10%. This concentration was used for all subsequent experiments to ensure minimal nonspecific interactions.

**Figure 5:**
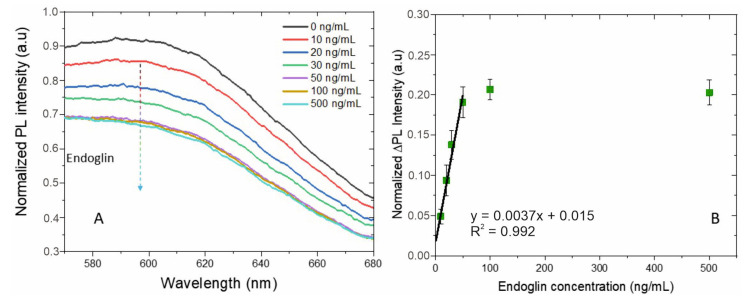
(**A**) PL intensity response of the biosensor to endoglin in the range from 10 ng/mL to 500 ng/mL and (**B**) Quenched PL intensity as a function of the endoglin concentration showing a linear range from 10 ng/mL to 50 ng/mL and saturation for values > 100 ng/mL.

The biosensor’s performance was assessed across a range of endoglin concentrations (10 ng/mL, 20 ng/mL, 30 ng/mL, 50 ng/mL, 100 ng/mL, and 500 ng/mL) (Figure 5A). In our system, when endoglin binds to the antibody-labeled AuNCs, it positions the protein acceptor in close proximity to the AuNCs (within a few nanometers), facilitating non-radiative energy transfer and resulting in photoluminescence quenching [63,64]. The observed PL quenching was usually attributed to by the effect of Föster or fluorescence resonance energy transfer (FRET) process, where energy from the excited state of the AuNCs is non-radiatively transferred to the endoglin protein-antibody complex, reducing the emission intensity [65,66,67,68]. The systematic correlation between decreasing PL intensity and increasing endoglin concentration supports the FRET mechanism. All sensing experiments were carried out in triplicates Figure S5. The PL response was measured using a fluorimeter, with excitation at 350 nm and maximum emission observed at 600 nm, a characteristic emission wavelength for AuNCs due to their quantum size effects [69,70].

A detailed analysis of the quenched PL intensity as a function of endoglin concentration (Figure 5B) revealed a linear relationship at lower concentrations (<50 ng/mL), followed by saturation at higher concentrations (>100 ng/mL). This saturation behavior suggests that at higher endoglin concentrations, most of the available antibody sites are occupied, leading to a plateau in the quenching response Figure 5A. The linear regression equation, PL (a.u.) = 0.0037 CEndoglin (ng/mL) + 0.015, exhibits a substantial correlation coefficient of (R^2^ = 0.992), with a limit of detection (LOD) of 5.4 ng/mL, calculated using the equation LOD = 3σ/s, where σ and s represent the standard deviation of the y-intercept and the linear regression slope respectively. The sensitivity of the biosensor, determined to be 0.004 a.u./[ng/mL], reflects the system’s ability to detect even minimal changes in endoglin concentration, underscoring its efficacy for early-stage detection of preeclampsia.

### 3.5. Comparative Analysis of Biosensor Performance

The analytical performance of the proposed hybrid AuNCs-NAA biosensor was compared with other biosensing platforms used in preeclampsia detection, as summarized in Table 1. Notably, this study represents the first application of endoglin as a sensing element specifically for preeclampsia detection, underscoring the novelty of this approach.

The hybrid AuNCs-NAA biosensor demonstrated a linear detection range for endoglin between 10 and 50 ng/mL, with a limit of detection (LOD) of 5.4 ng/mL and a sensitivity of 0.004 a.u./(ng/mL). These performance metrics are competitive with, and in some instances surpass, those reported for other biomarkers in preeclampsia-related studies. For instance, Estradiol (E2) detection using nanoporous microneedles exhibited a linear range of 0.1–1000 ng/mL (R^2^ = 0.926) [16], hile a surface-enhanced Raman scattering (SERS)-active gold nanoparticle system achieved a detection range of 0.1 nM to 100 nM for microRNA-20a with a correlation coefficient of R^2^ = 0.987 [76]. Additionally, lanthanide-doped upconversion nanoparticles conjugated with antibodies targeting FKBPL and CD44 showed a detection range of 1 ng/mL to nearly 100 ng/mL, with correlation coefficients of R^2^ = 0.998 for FKBPL and R^2^ = 0.994 for CD44 [74]. Other notable platforms include a plasmonic biosensor for CD81 detection, which covered a range of 0.1–1000 pg/mL with an LOD of 0.152 pg/mL [15], and a nanoparticle-enabled immunoassay integrated with an electrochemical plate, which quantified podocin and nephrin expression in urine with linear ranges of 0.05 to 500 ng/mL and LODs of 10.6 pg/mL for podocin (R^2^ = 0.978) and 14.5 pg/mL for nephrin (R^2^ = 0.989) [77].

The performance of any biosensor is highly dependent on the selected biomarker, leading to variations in detection limits and ranges across different platforms. Importantly, endoglin levels are known to be elevated months before the clinical onset of preeclampsia [78], making it a particularly valuable biomarker for early detection. Previous studies have developed enzyme-based diagnostic platforms capable of detecting endoglin in blood circulation with detection limits as low as 4.15 ng/mL [79]. Our biosensor aligns with these findings, demonstrating robust performance and sensitivity that are crucial for early diagnosis. Beyond its sensitivity, the hybrid AuNCs-NAA biosensor offers several practical advantages, including remote operability, cost-effectiveness, ease of use, and rapid detection within approximately 20 min. These features make it particularly well-suited for point-of-care diagnostics, where timely and accurate results are essential. Furthermore, the biosensor’s performance could be further enhanced by optimizing the geometrical properties of the NAA substrate, such as pore diameter, porosity, and thickness. Fine-tuning these parameters could increase the surface area available for biomolecule interaction, thereby improving detection sensitivity and overall biosensor performance. This foundational work lays the groundwork for future validation studies, where the biosensor’s performance in detecting endoglin will be assessed in the more complex matrix of real biological samples, further advancing its potential as a diagnostic tool.

### 3.6. Selectivity of the Biosensor

**Figure 6:**
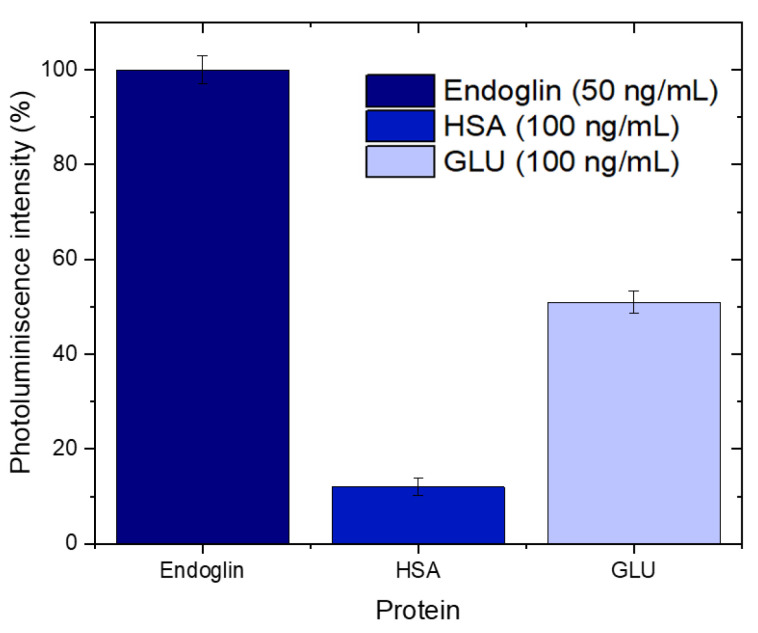
Selectivity test of the biosensor using two different proteins as a control, i.e., human serum albumin and glucoxidase.

The selectivity of the hybrid AuNCs-NAA biosensor was thoroughly evaluated to assess its specificity for endoglin detection in the presence of potentially interfering proteins. To this end, human serum albumin (HSA) and glucose oxidase (GLU) were selected as control proteins due to their common presence in biological samples, which could pose challenges for non-specific binding. The biosensor was tested with these proteins at concentrations twice that of endoglin (100 ng/mL for HSA and GLU compared to 50 ng/mL for endoglin). The proteins were incubated on the functionalized NAA surface for 15 min, followed by a 5-min PBS wash to remove any unbound proteins. The results, depicted in Figure 6, reveal a differential photoluminescence (PL) quenching response among the tested proteins. Specifically, the biosensor exhibited a 100% PL quenching response to endoglin, affirming its high affinity and specificity for this target biomarker. In contrast, GLU produced a 50% PL quenching response, indicating partial interaction with the biosensor, while HSA resulted in only a 10% quenching response, suggesting minimal non-specific binding. The 50% quenching response observed with GLU suggests that while the biosensor is highly specific to endoglin, there is some degree of non-specific interaction with GLU. However, this interaction is significantly less pronounced than that observed with endoglin, indicating that the biosensor can still reliably distinguish between endoglin and other proteins, though further optimization may be required to minimize this cross-reactivity. The minimal quenching observed with HSA underscores the biosensor’s ability to selectively target endoglin in the presence of highly abundant serum proteins, thereby reducing the likelihood of false positives.

## 4. Conclusions

This study presents a novel hybrid biosensor that integrates nanoporous anodic alumina (NAA) with gold nanoclusters (AuNCs) for the early diagnosis of preeclampsia. The functionalization of NAA substrates with APTES enabled the effective attachment of gold nanoclusters, creating a highly sensitive platform for biomarker detection. Endoglin, a critical biomarker associated with preeclampsia risk, was targeted, and the biosensor demonstrated a linear detection range from 10 to 50 ng/mL, encompassing the clinically relevant concentrations of endoglin. With a limit of detection (LOD) of 5.4 ng/mL, the biosensor effectively captures early changes in endoglin levels that are indicative of preeclampsia onset. The biosensor’s selectivity was rigorously validated, showing strong specificity for endoglin even in the presence of potentially interfering proteins, thus confirming its reliability for clinical applications. These results highlight the biosensor’s potential as a cost-effective, versatile, and portable tool for the early detection of preeclampsia, offering significant advantages for improving prenatal care and patient outcomes. To enable broader clinical application, future efforts could focus on miniaturization and integration with portable detection devices, enabling point-of-care diagnostics. Additionally, optimizing the geometrical properties of the NAA substrate, such as pore size and surface area, could further enhance sensitivity. Moreover, the optical biosensing platform developed in this study is not limited to preeclampsia diagnostics; its adaptable design allows for the detection of various biomarkers, opening avenues for broad applications in clinical diagnostics, food safety, and environmental monitoring. The demonstrated sensitivity, specificity, and adaptability of this biosensor underscore its potential to contribute to the development of next-generation diagnostic technologies, with far-reaching implications across multiple fields.

## Figures and Tables

**Table 1:** A Comparison of Various Biomarkers Used in Preeclampsia-Related Studies. PIGF: Placental growth factor, E2: Estradiol, CD44: Antigen CD44 and Gene FKBPL.

Analyte	Mechanism	Linear Range	Risk of Preeclampsia	LOD	Sensitivity	Reference
E2	Functionalized microneedles	0.1–1000 ng/mL	<17.9 ng/mL	50 pg/mL	13.03 (a.u/[pg/mL])	[16,71]
FKBPL	Strip-based lateral flow assay	1–100 ng/mL	CD44/FKBPL > 3.9	10 pg/mL	94,647 (a.u)	[72,73]
CD44				15 pg/mL	43,030 (a.u)	
PIGF	Nanoscale field transistors	0.1–1000 pg/mL	<100 pg/mL	0.06 pg/mL	0.77 pg/mL	[74,75]
Endoglin	Photoluminescence	10–50 ng/mL	>10 ng/mL	5.4 ng/mL	0.004 (a.u/[ng/mL])	This work

## Data Availability

The data that support the findings of this study are available from the corresponding author upon reasonable request.

## Supplementary Materials

The following supporting information can be downloaded at: https://www.mdpi.com/article/10.3390/bios14120610/s1, Supplementary material about the increment of the pore diameter, including additional FESEM images, EDX analysis of the NAA functionalized samples with gold nanoclusters, and PL spectra of the optimization of the gold nanoclusters binding to NAA. (PDF). Figure S1: NAA Samples with the AuNCs solution; Figure S2: Optimization of AuNCs binding; Figure S3: NAA FESEM images after second anodization; Figure S4: NAA FESEM top-view after pore widening and cross-sectional view; Figure S5: PL response of the sensor to different concentrations of Endoglin.
