# Supplementary material for: Sensing Platform Based on Gold Nanoclusters and Nanoporous Anodic Alumina for Preeclampsia Detection
Source: Biosensors (Basel). 2024 Dec 13;14(12):610. doi: 10.3390/bios14120610 (PMC11674843; doi:10.3390/bios14120610)
Supplement: Supplementary file 1 [file biosensors-14-00610-s001.zip › biosensors-3335424-supplementary.pdf]

Supporting Information

Sensing platform based on gold nanoclusters and nanoporous anodic alumina for preeclampsia detection

Josep Maria Cantons[1], Akash Bachhuka[1,2]*, and Lluis F. Marsal[1]*

[1]Department of Electronic, Electric and Automatics Engineering, Rovira i Virgili University, Tarragona 43007, Spain

[2] Institute of Chemical Research of Catalonia (ICIQ), Tarragona, Spain 43007.

*E-mails: akash.bachhuka@urv.cat, lluis.marsal@urv.cat

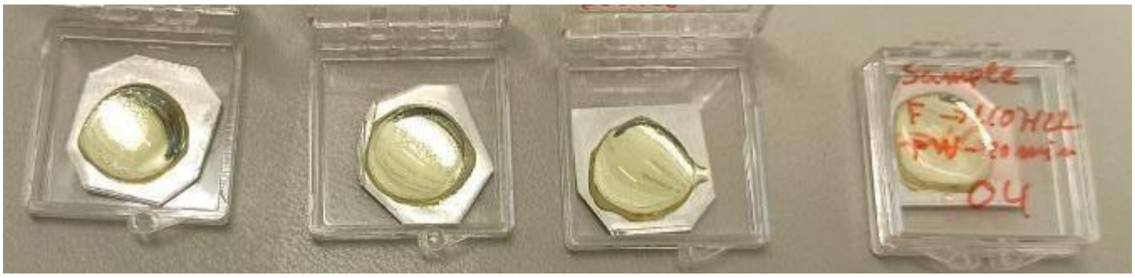

Figure S1: NAA samples with the AuNCs solution.

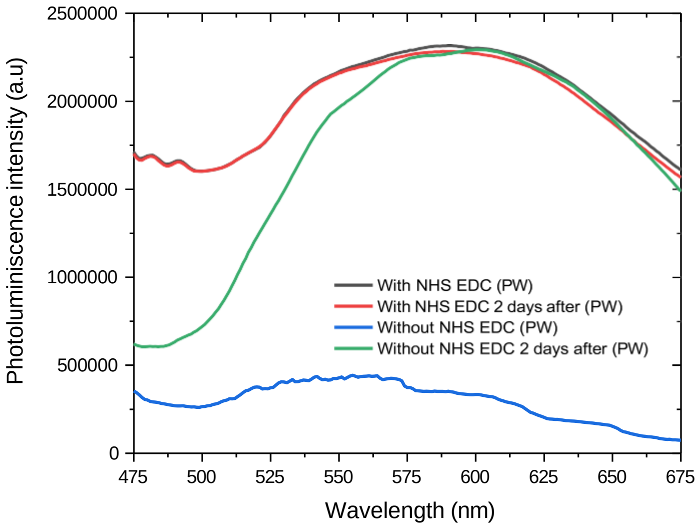

Figure S2: PL spectra for the optimization of AuNCs binding on APTES modified 65-75 nm pore size NAA samples.

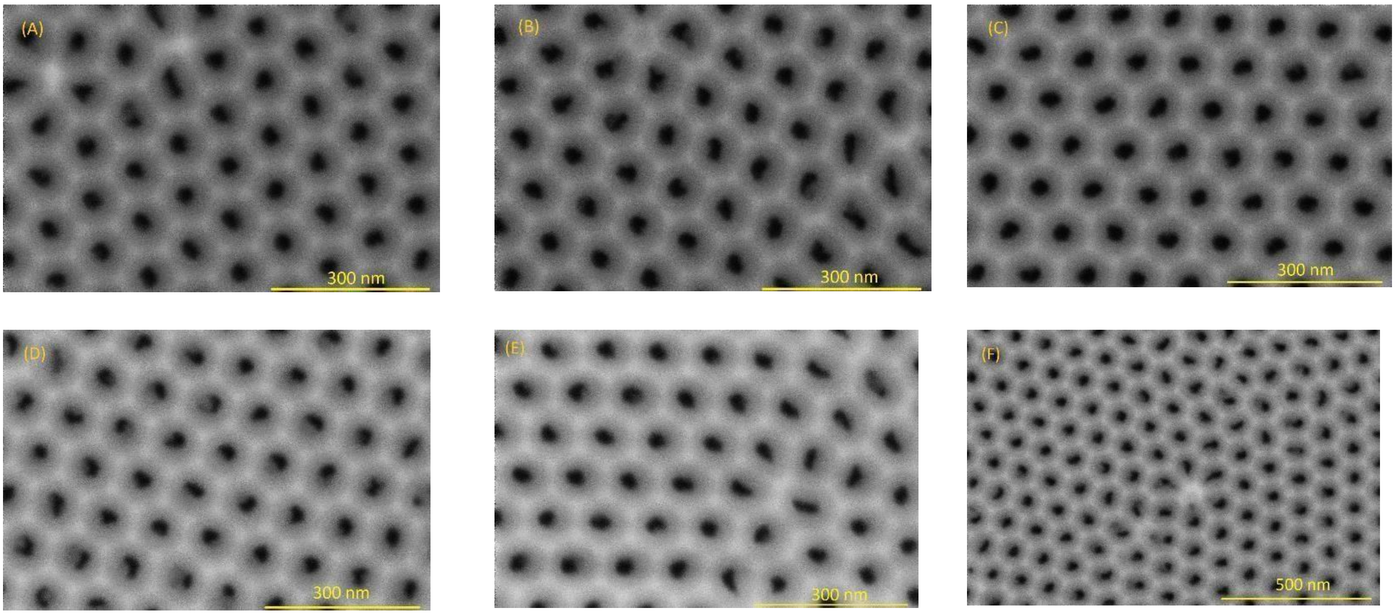

Figure S3: Top view FESEM images of NAA samples after second anodization. A) 29 nm average pore size, b) 33 nm average pore size, c) 30 nm average pore size, d) 31 nm average pore size, e) 33 nm average pore size and f) 32 nm average pore size. In these images it is shown that after the two-two step anodization with oxalic acid at 5 ºC and 40

V we obtain pore sizes from 30 nm to 35 nm.

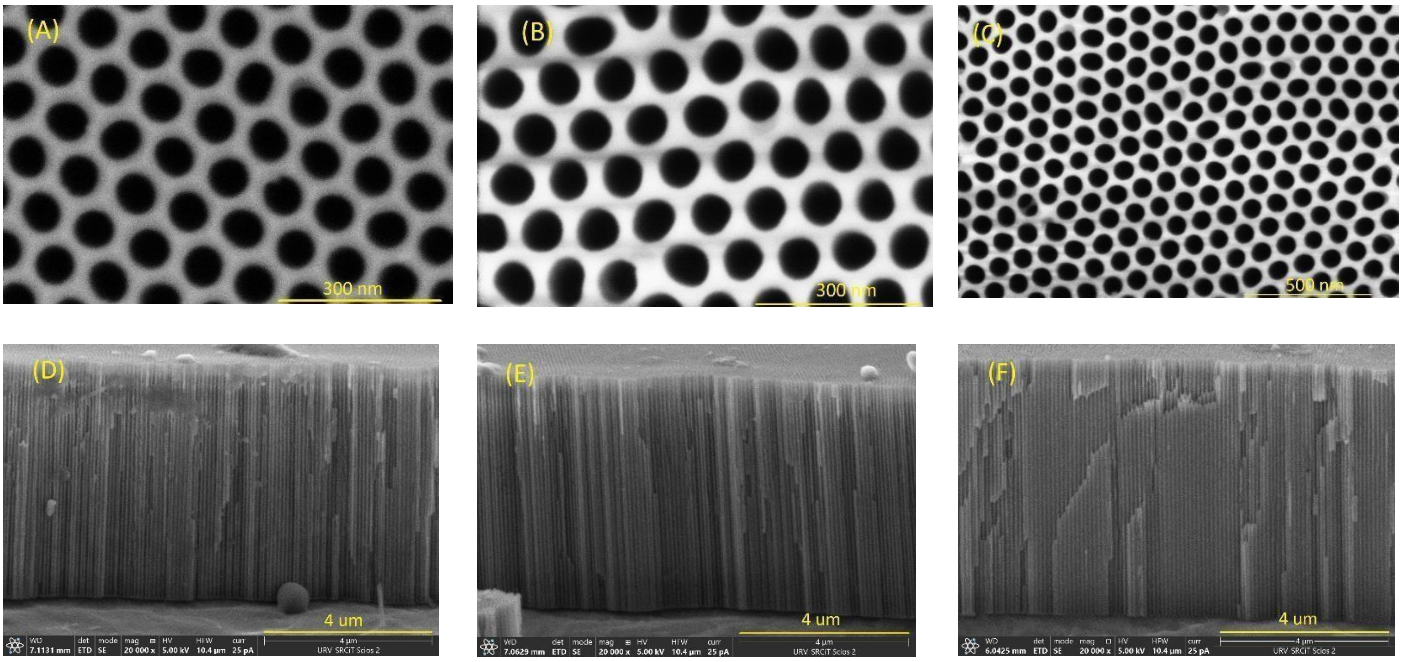

Figure S4: Top view of NAA samples after 22 minutes of pore widening and cross section of NAA samples after a second anodization (95 C). A) 65 nm average pore size, b) 69 nm average pore size, c) 69 nm average pore size, d) 5.7 µm average thickness, e) 5.8 µm average thickness and f) 6.1 µm average thickness. The first three images show that after 22 minutes of pore widening, we can obtain pore sizes higher than 65 nm, and the three below show that after applying 95 C on the second anodization we obtain approximately 6 µm of pore length.

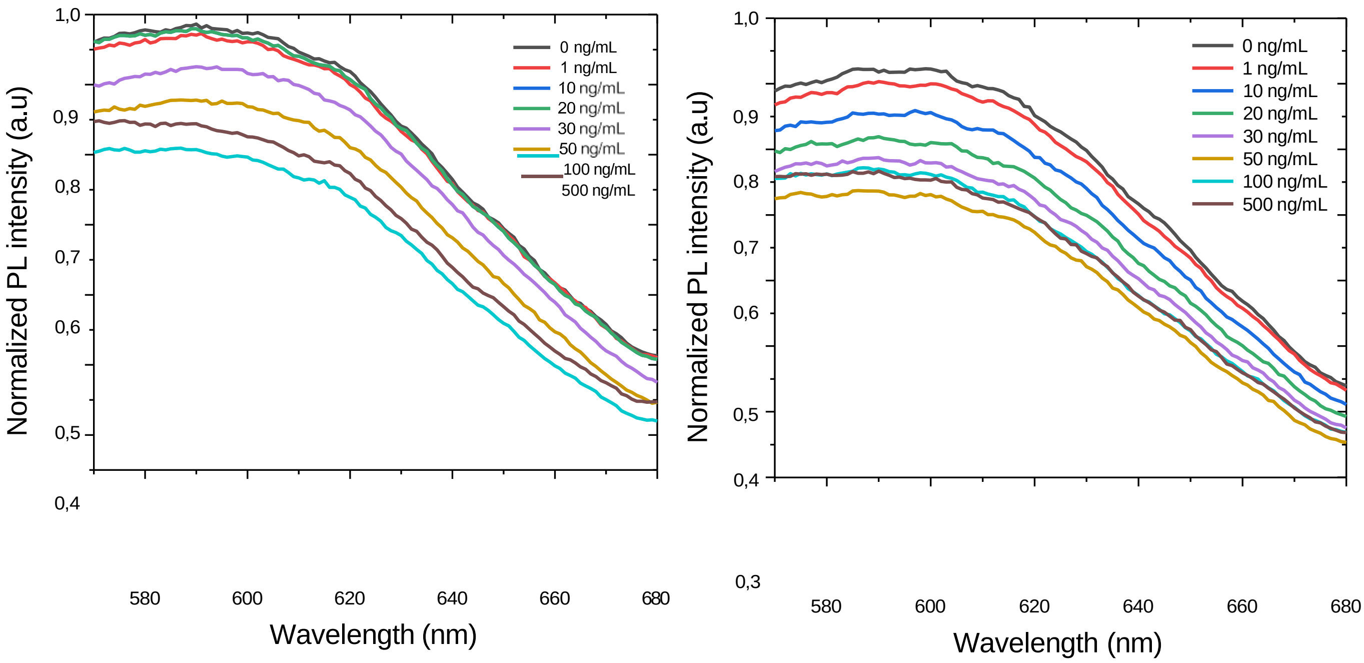

Figure S5: PL response of two of the sensors using concentrations of Endoglin in a range that covers its presence in Preeclampsia disease. The sensor shows a quenching in the photoluminescence in both experiments as we increased the Endoglin concentrations.
